# Pd(II) and Pt(II) Complexes of Schiff Thiobases Derived From 2-Carbonylpyridine

**DOI:** 10.1155/MBD.1998.161

**Published:** 1998

**Authors:** M. Gómez-Bosquet, V. Moreno, M. Font-Bardía, X. Solans

**Affiliations:** 1 Departament de Química Inorgànica Universitat de Barcelona Avgda Diagonal, 647 Barcelona 08028 Spain; 2 Departament de Cristallografia Dipòsits Minerals Martí i Franquès s/n Barcelona 08028 Spain

## Abstract

Pd(ll) and Pt(ll) complexes of three series of Schiff thiobases derived from 2- carbonylpyridine have been synthesized and characterized. The crystal structure of the Pt(ll) derivative of methyl-3-(2-pyridylmethylene)hydrazinecarbodithioate (HFp) was resolved. The ligand coordinates the platinum ion in tridentate fashion by heterocycle and imine nitrogen and thiocarbonyl sulfur. The fourth ligand is a chloride ion. The structure of the complexes is suitable for the formation of monofunctional adducts with DNA. Studies on the interaction of the complexes with *Calf thymus* DNA by CD reveal modifications in the B form of lineal DNA. Interaction with plasmid DNA was also confirmed in the images obtained by atomic force microscopy.


### Pd(II) AND Pt(II) COMPLEXES OF SCHIFF THIOBASES DERIVED FROM 2-CARBONYLPYRIDINE

M. Gómez-Bosquet[1], V. Moreno*[1], M. Font-Bardía[2] and X. Solans[2]

[1] Departament de Química Inorgànica, Universitat de Barcelona, Avgda Diagonal, 647, 08028 Barcelona, Spain

[2] Departament de Cristallografia i Dipòsits Minerals, Martí i Franquès s/n, 08028 Barcelona, Spain

### ABSTRACT

Pd(II) and Pt(II) complexes of three series of Schiff thiobases derived from 2-carbonylpyridine have been synthesized and characterized. The crystal structure of the Pt(II) derivative of methyl-3-(2-pyridylmethylene)hydrazinecarbodithioate (HFp) was resolved. The ligand coordinates the platinum ion in tridentate fashion by heterocycle and imine nitrogen and thiocarbonyl sulfur. The fourth ligand is a chloride ion. The structure of the complexes is suitable for the formation of monofunctional adducts with DNA. Studies on the interaction of the complexes with *Calf thymus* DNA by CD reveal modifications in the B form of lineal DNA. Interaction with plasmid DNA was also confirmed in the images obtained by atomic force microscopy.

### INTRODUCTION

In recent decades many complexes of transition metal containing an S donor atom have been described as antimalarial, antileukemic or antiviral agents[1-7]. The pyridine derivatives of thiosemicarbazones have been widely studied. Copper(II), zinc(II), cadmium(II), nickel(II), iron(III), manganese(II) and cobalt(III) were described as possible enhancers of the antileukemic properties by coordination to ligands of this kind[8-19]. Palladium and platinum complexes of dithiocarbazic acid and its derivatives were described[20-22]. Recently, the crystal structures of palladium(II) complexes of 2-acetylpyridine N(4)-dimethylthiosemicarbazone have been reported[23-24]. The presence of only one halide ion in the coordination sphere of the metal enables the molecule to form monofunctional adducts with nucleotides or DNA and thus can explain the possible antitumour or antileukemic activity of the analogous platinum compounds. We have synthesized, characterized and studied the interaction with *Calf thymus* DNA and plasmid pBR322 DNA of three series of Pd(II) and Pt(II) compounds with the Schiff bases HAp (the 2-acetylpyridine Schiff base of S-methyldithiocarbazate), HBp (the 2-benzoylpyridine Schiff base of S-methyldithiocarbazate) and HFp (the 2-formylpyridine Schiff base of S-methyldithiocarbazate). The resolution of the crystal structure of one of the Pt(II) compounds, the 2-formylpyridine Schiff base of S-methyldithiocarbazate, has confirmed that the molecular structure is analogous to that of palladium complexes reported in the literature.

### EXPERIMENTAL

### *Materials and Methods*

The complexes were prepared using Johnson Matthey $K_2[PdCl_4]$ and $K_2[PtCl_4]$, Aldrich 2-acetyl, 2-benzoyl and 2-formylpyridine and Carlo Erba $CS_2$ and $CH_3I$. Elemental analyses were carried out on a Carlo Erba 1500 microanalyzer at the Serveis Científico-Tècnics at the University of Barcelona. The infrared spectra were recorded in solid state (KBr pellets) on an FT-IR Nicolet 5DZ spectrometer in the 4000-400 $cm^{-1}$ range. $^1H\{^{13}C\}$, $^{13}C\{^1H\}$ and $^{195}Pt\{^1H\}$NMR spectra were obtained on a Brucker DRX 250 spectrometer using DMSO-$d_6$ as solvent. Chemical shifts were measured relative to TMS in the case of $^1H$ and $^{13}C$ and to $Na_2PtCl_6$ for $^{195}Pt$.

CD spectra were obtained on a JASCO J720 spectropolarimeter with a 450 W xenon lamp.

Images of plasmid pBR322 DNA, and adducts of Pt compounds with this DNA were obtained with Extended Nanoscope III (Digital Instruments, Santa Barbara, CA) working in TMAFM mode in about 100 nN. The samples were prepared by incubating the complexes dissolved in a DMSO-Hepes (30:70) mixture with plasmid pBR322 DNA at 37°C for 24 hour at several molar ratios complex/DNA (ri). A drop of 2 µl of DNA or DNA-metal complex solution was deposited onto freshly cleaved green mica (Ashville-Schoonmaker Mica Co., Newport New, VA). After adsorption for five minutes at room temperature, the samples were rinsed for ten seconds in a jet of deionized water of

18 MWcm$^{-1}$ from a Milli-Q water purification system (Millipore, Molshem, France) directed onto the surface with a squeeze bottle. The samples were blow dried with compressed argon over silica gel for 30 minutes before imaging in the AFM.

### *Synthesis of the ligands*

All the ligands were prepared by the same general method[12]: S-methyldithiocarbazate[22] (10 mmol) in ethanol (40 cm$^3$) was mixed with a solution of 2-acetyl, 2-benzoyl or 2-formylpyridine in the same solvent. The reaction mixture was boiled on a waterbath and its volume was reduced to 30 cm$^3$. The product that formed was collected and dried *in vacuo* over silica gel. The ligands synthesized were HAp (the 2-acetylpyridine Schiff base of S-methyldithiocarbazate), HBp (the 2-benzoylpyridine Schiff base of S-methyldithiocarbazate) and HFp (the 2-formylpyridine Schiff base of S-methyldithiocarbazate).

### *Synthesis of Pd(II) and Pt(II) complexes*

The metal complexes were prepared by the same general procedure. A solution of $K_2PtCl_4$ or $K_2PdCl_4$ (1 mmol) in $H_2O$ (20 cm$^3$) was mixed with a solution of the ligand (1 mmol) in ethanol (20 cm$^3$). The mixture was boiled on a waterbath for ca 20 min and then left to cool. The product that deposited was filtered off, washed in ethanol and dried *in vacuo* over silica gel.

Table 1: Colours, molar conductivities and analytical data.

| Compound | Found (Calc.) % | Colour | $\lambda_M$$^a$ |
|---|---|---|---|
| **HAp** | C-48.47 (48.47) N-18.73 (18.65)<br>H-5.24 (4.92) S-28.27 (28.45) | Yellow | 0.96 |
| **PtAp** | C-23.65 (23.76) N-9.44 (9.24)<br>H-2.50 (2.22) S-14.22 (14.10) Cl-8.14 (7.79) | Red | 4.23 |
| **PdAp** | C-30.96 (29.52) N-11.50 (11.48)<br>H-3.01 (2.75) S-17.51 (17.51) Cl-9.81 (9.68) | Orange | 1.25 |
| **HBp** | C-58.70 (58.51) N-14.79 (14.62)<br>H-4.58 (4.56) S-22.41 (22.31) | Yellow | 0.68 |
| **PtBp** | C-32.72 (32.53) N-8.33 (8.13)<br>H-2.42 (2.73) S-12.32 (12.40) Cl-6.12 (6.86) | Red brown | 8.67 |
| **PdBp** | C-38.80 (39.26) N-9.78 (9.81)<br>H-2.78 (2.82) S-14.82 (14.97) Cl-7.89 (8.28) | Light brown | 4.16 |
| **HFp** | C-45.83 (45.48) N-19.84 (19.89)<br>H-4.51 (4.29) S-29.92 (30.34) | Light yellow | 0.29 |
| **PtFp** | C-21.12 (21.79) N-8.82 (9.50)<br>H-1.99 (1.83) S-14.07 (14.54) Cl-8.26 (7.79) | Red | 4.30 |
| **PdFp** | C-27.58 (27.29) N-11.66 (11.93)<br>H-2.49 (2.29) S-17.87 (18.21) Cl-9.78 (10.07) | Orange | 2.64 |

a: $\Omega^{-1}cm^2mol^{-1}$ in a solution of DMF $10^{-3}$M

### *X-ray diffraction*

A prismatic crystal (0.1x0.1x0.2 mm) of PtFp was selected and mounted on an Enraf-Nonius CAD4 four-circle diffractometer. Unit cell parameters were determined from automatic centering of 25 reflections (12<Θ<21º) and refined by least-squares method. Intensities were collected with graphite monochromatized Mo$K_\alpha$ radiation, using $\omega/2\Theta$ scan technique. 2261 reflections were measured in the range $2.39 \leq \Theta \leq 29.98$, 2161 of which were non-equivalent by symmetry (Rint(on I) = 0.022). 1747 reflections were assumed as observed applying the condition $I>2\sigma(I)$. Three reflections were measured every two hours as orientation and intensity control, significant intensity decay was not observed. Lorentz-polarization and absorption corrections were applied[25].

The structure has a monoclinic cell, with cell parameters a = 9.357 Å; b = 9.408 Å and c = 13.431 Å. The structure was solved by direct methods using SHELXS computer program[26] and refined by full-matrix least-squares method with SHELX93 computer program[27] (very negative intensities were not assumed). The function minimized was $S\omega[(Fo)^2 - (Fc)^2]^2$, where $w = [s^2(I) + (0.0385\ P)^2\ )^{-1}$, and $P = [(Fo)^2 + 2\ (Fc)^2]/3$, f, f' and f" were taken from International Tables of X-Ray Crystallography[28]. All the H atoms were located from a difference synthesis, all were refined with an overall isotropic temperature factor, using a riding model. The final R (on F) factor was 0.0360, wR [on $(F)^2$] = 0.0856. Crystal data and summary of data collection are presented in Table 1.

**Table 1.** Crystal data and Summary of Date collection and refinement for PtFp.

| | | | |
|---|---|---|---|
| **Formula** | $C_8H_8ClN_3S_2Pt$ | **Absorption coeff.** | 12.846 $mm^{-1}$ |
| **Mol. weight** | 440.83 | **Density (calc.)** | 2.562 $g/cm^3$ |
| **Crystal system** | Monoclinic | **F(000)** | 816 |
| **Space group** | $P2_1/n$ | **Temperature** | 293(2) K |
| **a** | 9.357(3) Å | **Crystal size** | 0.1x0.1x0.2 mm |
| **b** | 9.408(9) Å | **Radiation** | MoKα (λ=0.71096 Å) |
| **c** | 13.431(7) Å | **Data coll. range θ** | $2.39 \leq \theta \leq 29.98$ º |
| α | 90 ° | **N. of measured refl.** | 2261 |
| β | 104.88(3) ° | **N. of unique refl.** | 2161 |
| γ | 90 ° | **N. of ob. ref. [I>2σ(I)]** | 1747 |
| **V** | 1142.7(13) $Å^3$ | **Final R** | R1=0.0360, wR2=0.0856 |
| **Z** | 4 | **$R_{all\ the\ data}$** | R1=0.0499, wR2=0.0897 |

Final atomic coordinates are collected in Table 2. Hydrogen coordinates as well as anisotropic thermal parameters are included as supplementary material.

**Table 2.** Atomic coordinates ( x $10^4$) and equivalent isotropic displacement parameters ($Å^2 x 10^3$) for 1. U(eq) is defined as one third of the trace of the orthogonalized $U_{ij}$ tensor.

| | x | y | z | U(eq) |
|---|---|---|---|---|
| Pt | 1698(1) | 9759(1) | 1027(1) | 36(1) |
| Cl | 3262(4) | 8551(3) | 261(3) | 63(1) |
| S(1) | 789(3) | 7830(3) | 1627(2) | 41(1) |
| S(2) | -1438(4) | 7917(4) | 2930(3) | 58(1) |
| N(2) | 389(10) | 10846(11) | 1777(7) | 44(3) |
| N(3) | -411(11) | 10133(13) | 2293(8) | 57(3) |
| N(1) | 2150(11) | 11791(11) | 614(8) | 51(3) |
| C(1) | 3057(15) | 12208(14) | 21(9) | 50(3) |
| C(2) | 3242(14) | 13678(14) | -134(10) | 55(3) |
| C(3) | 2543(15) | 14658(15) | 310(10) | 55(3) |
| C(4) | 1665(16) | 14226(14) | 907(10) | 58(4) |
| C(5) | 1431(14) | 12777(14) | 1070(10) | 52(4) |
| C(6) | 558(15) | 12192(11) | 1679(10) | 72(4) |
| C(7) | -254(13) | 8839(15) | 2242(9) | 47(3) |
| C(8) | -1063(15) | 6104(12) | 2873(9) | 58(4) |

### RESULTS AND DISCUSSION

### *Spectral studies*

The main vibrational bands of the ligands and their complexes are reported in Table 3.

**Table 3**: Selected IR absorption frequencies ($cm^{-1}$) for the ligands and their Pt(II) and Pd(II) complexes.

| Compound | Thioamide I | Thioamide II | Thioamide III | ν(C=N) | ν(N-N) | ν(M-N) | ν(M-S) |
|---|---|---|---|---|---|---|---|
| HAp | 1462 | 1264 | 948 | 1581 | 1060 | — | — |
| PtAp | 1448 | — | 955 | 1602 | 1047 | 429 | 352 |
| PdAp | 1448 | — | 955 | 1595 | 1040 | 420 | 361 |
| HBp | 1469 | 1244 | 948 | 1581 | 1054 | — | — |
| PtBp | 1427 | — | 956 | 1595 | 998 | 434 | 339 |
| PdBp | 1427 | — | 955 | 1595 | 998 | 430 | 337 |
| HFp | 1469 | 1279 | 998 | 1581 | 1047 | — | — |
| PtFp | 1398 | — | 1005 | 1602 | 1061 | 425 | 351 |
| PdFp | 1434 | — | 991 | 1595 | 1061 | 412 | 361 |

The Schiff bases contain the thioamide group and consequently may participate in a thione-thiol tautomerism. The i.r. spectra of the ligands do not show the ν(S-H) band at ca 2565 $cm^{-1}$, but they do show the ν(N-H) band at ca 3150 $cm^{-1}$, indicating that, in the solid state, they are probably in the thione form. However, in the presence of the metal ion, the tautomerization to the thiol and deprotonation is enhanced, and the ligands coordinate in the deprotonated form, as do related thiosemicarbazone ligands. The i.r. spectra of the metal(II) complexes do not contain either the ν(N-H) or the ν(S-H) bands, supporting the coordination with the singly negatively charged ligand.

An unambiguous assignment of the ν(C=S) is only possible when this group is not linked to a N atom because of the vibrational coupling effects[29]. In the thioamide system, coupling can take place among C-N stretching, C=S stretching and NH deformation vibrations, and the C=S vibration is not localized. However three bands seem to appear in the regions 1395-1570, 1260-1420 and 940-1140 $cm^{-1}$ due to the mixed vibrations. The shift of two of the thioamide bands and the disappearance of the third support the deprotonation during the coordination. This third band, the thioamide II, seems to have a significant contribution of the C=S stretching.

The azomethine bands in the i.r. spectra of the complexes appear in a range 1595-1602 $cm^{-1}$, somewhat higher than those observed for the free ligands. The bands corresponding to the two possible N=C bonds are not resolved in the spectra of these complexes. Some related compounds show a positive shift[30,31], but others show a negative shift[32,33] owing to the fact that they are combination bands, especially in pyridine compounds[8]. The coordination through the azomethine nitrogen atom is also consistent with the shift of the ν(N-N) bands.

The presence of ν(M-N) and ν(M-S) bands also supports the coordination via the azomethine and pyridine nitrogen atom, and the mercaptide sulfur atom.

The proton NMR spectra of the Schiff bases in $d_6$-DMSO do not show any peak near 4 ppm attributable to an S-H proton, but they do show the peak of the N-H proton. In all cases these peaks appear downfield shifted (12-15 ppm) compared with a typical proton of a secondary amine (9 ppm), and they are closer to a protonated heterocyclic nitrogen. Two of the isomers of the ligands in solution show an interaction between the N-H proton and the pyridine nitrogen, although a similar shift could also be caused by an interaction of the H with DMSO. This behaviour has also been observed in similar thiosemicarbazones[19]. This peak disappears in the spectra of the complexes, which supports the coordination through the thiol form of the ligand.

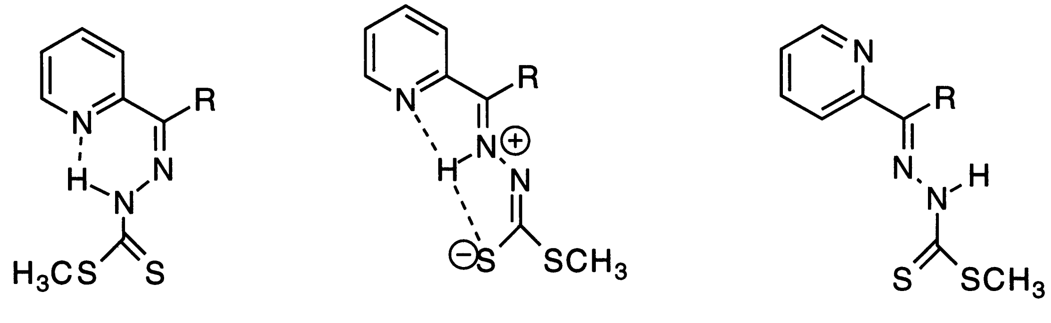

Z E' E

**Table 4.** $^1H$ and $^{13}C$ NMR data (δ, in ppm) for the ligands and complexes synthesized.

| Compound | NH | C7 | C6 | C1 |
|---|---|---|---|---|
| HAp | 12.57 | 201.11 | 154.73 | 152.15 |
| PtAp | — | a | a | a |
| PdAp | — | a | a | a |
| HBp | 14.42 | 200.80 | 151.33 | 149.51 |
| PtBp | — | 194.81 | 164.50 | 160.53 |
| PdBp | — | 192.34 | 163.23 | 159.43 |
| HFp | 13.46 | 199.76 | 152.75 | 150.18 |
| PtFp | — | 195.59 | 157.35 | 159.42 |
| PdFp | — | 192.69 | 155.99 | 157.51 |

a: not available.

The $^{13}C$-NMR spectra of the complexes show an upfield shift of the C=S peak (C7), and downfield shifts of the C=N (azomethine carbon atom, C6) and of the C close to the pyridinic N (C1), according to the coordination through the NNS system. In all the compounds $d_6$-DMSO was used as solvent, but the spectra of the complexes of the HAp ligand were not available due to insufficient solubility.

The $^{195}Pt$-NMR spectra of the complexes PtBp and PtFp show a shift corresponding to a NNS coordination (again the PtAp spectrum was not available). The signals appear at -3207 and -3208 ppm referred to $K_2PtCl_6$.

The mass spectra of the complexes, registered in acetonitrile, show the same pattern in all cases, consistent with the monomeric formulation of the compounds. The strongest peak is due to the $[ML(CH_3CN)]^+$ ion, in which the lost Cl atom has been replaced by a molecule of the solvent. This substitution has been observed in many molecules[34]. A peak attributable to the $[(ML)_2(Cl)]^+$ ion has also been observed.

**Table 5**. Mass spectroscopic data (m/z) for the complexes synthesized.

| Compound | $[ML(CH_3CN)]^+$ | $[(ML)_2(Cl)]^+$ |
|---|---|---|
| PtAp | 460 | a |
| PdAp | 373 | 697 |
| PtBp | 522 | 998 |
| PdBp | 433 | 822 |
| PtFp | 446 | 846 |
| PdFp | 359 | 669 |

L = $Ap^-$, $Bp^-$, $Fp^-$ a: not available

***Crystal and molecular structure of chloro(S-methyl-β-N-(2-pyridyl)dithiocarbazate) platinum(II)***

In Figure 1 the molecular structure of the complex chloro(S-methyl-β-N-(2-pyridyl)dithiocarbazate)platinum(II) is shown. Bond distances and angles are collected in Table 6 .

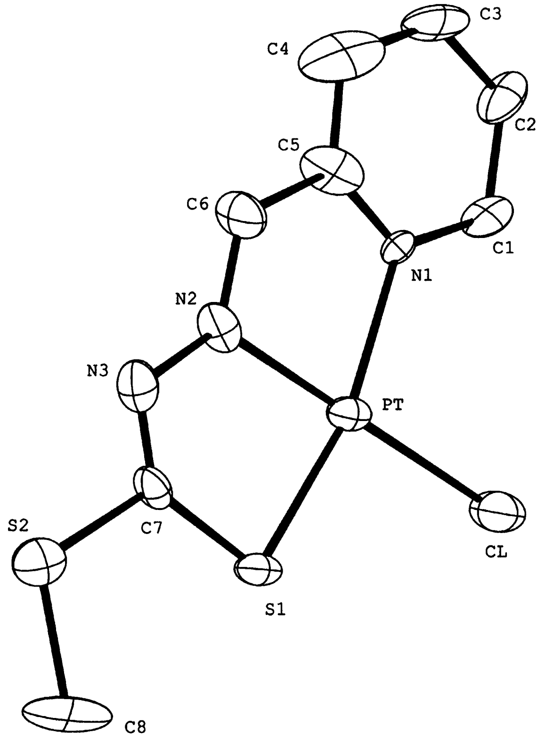

Figure1. Molecular structure of chloro-(S-methyl -β-N-(2-pyridyl)dithiocarbazate)platinum(II)

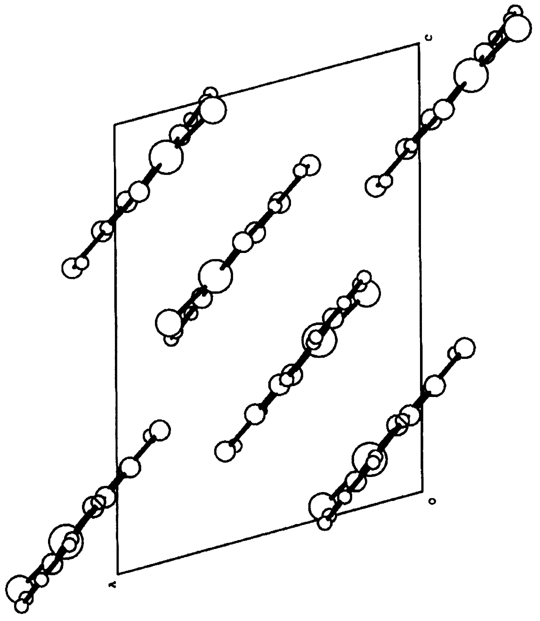

Figure 2: Monoclinic cell with four molecules of the complex

The platinum atom is localized on a plane square. The thiocarbazate ligand coordinates in tridentate fashion to the pyridinic nitrogen N(1), the azomethinic nitrogen N(2) and the thiolate sulfur S(1). The fourth coordination site is occupied by a chlorine atom.

The Schiff base is an anion with a single negative charge, which allows tautomerism to the iminothiolate form, as has been described for the analogous thiocarbazate[14] and thiosemicarbazone[15] complexes. So, the negative charge generated in mercaptane deprotonation is delocalized in the C-N-N-C system when the ligand binds to the platinum atom. This is reflected in the intermediate values of the bond distances : N(2)-C(6) = 1.287 Å, N(2)-N(3) = 1.327 Å, N(3)-C(7) = 1.23 Å. The bond distance S(1)-C(7) is 1.718 Å, which corresponds to a single bond, while the distances C-N and N-N are close to the values corresponding to double bonds. The bond distances for Pt-S, Pt-N(imine) and Pt-N(pyridine) are 2.241, 2.048 and 2.064 Å respectively. These values are consistent with the values found in the literature for analogous complexes[14, 15]. The angles N(1)-Pt-S(1) and N(2)-Pt-Cl are smaller than the ideal angle of 180°. The two rings of five members are almost plane. So, the planarity together with the values of the bond distances indicate an extended delocalization of the π electrons into the chelate rings.

The Figure 2 shows the monoclinic cell with four molecules of the complex. These are parallel to their symmetry plane, but alternated in reference to the chlorine atoms.

**Table 6.** Bond Lengths (Å) and Bond Angles (°) with their e.s.d.'s for chloro-3-(2-pyridylmethylidene)hydrazinemethylcarbodithioate platinum(II) (PtFp)

| **Bond lengths (Å)** | | | |
|---|---|---|---|
| **Pt-N(2)** | 2.048 (10) | **N(3)-C(7)** | 1.23 (2) |
| **Pt-N(1)** | 2.064 (10) | **N(1)-C(1)** | 1.36 (2) |
| **Pt-S(1)** | 2.241 (3) | **N(1)-C(5)** | 1.38 (2) |
| **Pt-Cl** | 2.295 (3) | **C(1)-C(2)** | 1.42 (2) |
| **S(1)-C(7)** | 1.718 (12) | **C(2)-C(3)** | 1.35 (2) |
| **S(2)-C(8)** | 1.747 (12) | **C(3)-C(4)** | 1.35 (2) |
| **S(2)-C(7)** | 1.833 (13) | **C(4)-C(5)** | 1.41 (2) |
| **N(2)-C(6)** | 1.287 (14) | **C(5)-C(6)** | 1.41 (2) |
| **N(2)-N(3)** | 1.327 (13) | | |
| **Bond angles (°)** | | | |
| **N(2)-Pt-N(1)** | 81.9 (5) | **C(1)-N(1)-Pt** | 128.7 (9) |
| **N(2)-Pt-S(1)** | 84.1 (3) | **C(5)-N(1)-Pt** | 110.3 (9) |
| **N(1)-Pt-S(1)** | 165.8 (3) | **N(1)-C(1)-C(2)** | 119.0 (12) |
| **N(2)-Pt-Cl** | 177.1 (3) | **C(4)-C(3)-C(2)** | 119.6 (13) |
| **N(1)-Pt-Cl** | 98.1 (3) | **C(3)-C(4)-C(5)** | 121.8 (14) |
| **S(1)-Pt-Cl** | 96.04 (13) | **N(1)-C(5)-C(6)** | 114.6 (11) |
| **C(7)-S(1)-Pt** | 92.3 (5) | **N(1)-C(5)-C(4)** | 118.1 (14) |
| **C(8)-S(2)-C(7)** | 106.6 (6) | **C(6)-C(5)-C(4)** | 127.3 (13) |
| **C(6)-N(2)-N(3)** | 130.7 (10) | **N(2)-C(6)-C(5)** | 123.3 (10) |
| **C(6)-N(2)-Pt** | 109.7 (8) | **N(3)-C(7)-S(1)** | 131.7 (11) |
| **N(3)-N(2)-Pt** | 119.6 (8) | **N(3)-C(7)-S(2)** | 110.1 (9) |

### *STUDY OF THE INTERACTION WITH DNA*

### *Circular Dichroism*

The circular dichroism spectra of *Calf Thymus* DNA and *Calf Thymus* DNA incubated with the complexes at 37° C for 24 h with several molar ratios were recorded. The $\Theta_{max}$ and $\Theta_{min}$ for $\lambda_{max}$ and $\lambda_{min}$ values at different molar ratio ri, are collected in tables 7, 8 and 9.

The ligands and the complexes showed a slight modification of the circular dichroism spectra. This behaviour has been observed in another compound with unique position of coordination, [Pt(dien)Cl]Cl[35,36]. However, the modification caused by HBp and its complexes is greater that the produced by HAp and HFp.

**Table 7:** CD of DNA/HAp compounds at different molar ratio after 24 h of incubation.

| Sample | ri | $\Theta_{max}{}^a$ | $\lambda_{max}{}^b$ | $\Theta_{min}{}^a$ | $\lambda_{min}{}^b$ |
|---|---|---|---|---|---|
| DNA | | 8.89 | 276.5 | -11.13 | 245.5 |
| HAp | 0.05 | 9.00 | 278.5 | -11.13 | 246.5 |
| | 0.10 | 9.05 | 274.0 | -10.78 | 246.0 |
| | 0.30 | 9.15 | 275.5 | -11.03 | 246.0 |
| | 0.50 | 9.17 | 277.0 | -10.78 | 247.0 |
| PtAp | 0.05 | 8.86 | 275.5 | -11.17 | 245.5 |
| | 0.10 | 9.05 | 273.0 | -10.21 | 242.0 |
| | 0.30 | 8.74 | 274.5 | -10.64 | 244.5 |
| | 0.50 | 8.56 | 278.5 | -10.69 | 246.5 |
| PdAp | 0.05 | 9.08 | 275.0 | -10.82 | 246.0 |
| | 0.10 | 9.05 | 277.0 | -10.55 | 245.5 |
| | 0.30 | 9.20 | 276.5 | -10.56 | 245.0 |
| | 0.50 | 8.73 | 276.5 | -11.18 | 247.0 |

a: degree.$cm^2.dmol^{-1}.10^3$
b: nm

**Table 8:** CD of DNA/HBp compounds at different molar ratio after 24 h incubation.

| Sample | ri | $\Theta_{max}{}^a$ | $\lambda_{max}{}^b$ | $\Theta_{min}{}^a$ | $\lambda_{min}{}^b$ |
|---|---|---|---|---|---|
| DNA | | 8.56 | 275.5 | -10.52 | 245.0 |
| HBp | 0.05 | 8.75 | 279.0 | -10.49 | 246.5 |
| | 0.10 | 8.95 | 277.5 | -10.71 | 246.5 |
| | 0.30 | 8.31 | 276.0 | -11.39 | 246.0 |
| | 0.50 | 8.29 | 274.5 | -10.35 | 245.5 |
| PtBp | 0.05 | 8.69 | 273.5 | -10.39 | 246.0 |
| | 0.10 | 8.26 | 278.5 | -10.75 | 246.0 |
| | 0.30 | 8.33 | 275.0 | -9.57 | 246.0 |
| | 0.50 | 8.25 | 280.0 | -9.77 | 246.0 |
| PdBp | 0.05 | 8.59 | 277.0 | -10.75 | 245.5 |
| | 0.10 | 8.75 | 275.5 | -10.47 | 246.5 |
| | 0.30 | 8.41 | 277.0 | -9.86 | 246.0 |
| | 0.50 | 7.60 | 280.0 | -8.55 | 248.0 |

a: degree.$cm^2.dmol^{-1}.10^3$
b: nm

The CD spectrum of the adduct DNA:ligand shows a slight increase in the ellipticity of the positive band, similar to that of Pd-complex. This behaviour may be due to modifications in the base stacking, which produce an increase in the coiling of the double helix and stabilization of the B form of DNA.

However, the Pt-complex shows a different behaviour, decreasing the ellipticity of this band for the same values of ri. This effect may be due to an instabilization of the B form of DNA.

In the CD spectra of the HBp series slightly higher differences in the ellipticity values can be detected, probably due to the presence of an R group in the β position in the pyridine ring of the HBp ligand. This group is larger than the methyl group of the HAp ligand or H of the HFp ligand and, due to its plane geometry, it can intercalate between the base-pairs of DNA causing a greater interaction. The behaviour observed for the ligand HBp and for its complexes is analogous to that of cisplatin. The ellipticity increases in the positive band for small values of ri and decreases when ri increases.

In this case, from the observation of the CD spectra, it can be deduced that the ligand HFp and its Pd complex show the same behaviour, stabilization of the B form of DNA, while the Pt complex causes an instabilization of this form. In conclusion, there is parallel in the interaction of HAp and HFp and their respective derivatives with DNA. The behaviour is similar to that of cisplatin. However, in the HBp series, the changes in the ellipticity of DNA are greater, probably due to the presence of the R group which could allow an additional interaction with the double helix.

**Figure 3:** Atomic Force Microscopy Images
**a)** plasmid pBR322 DNA

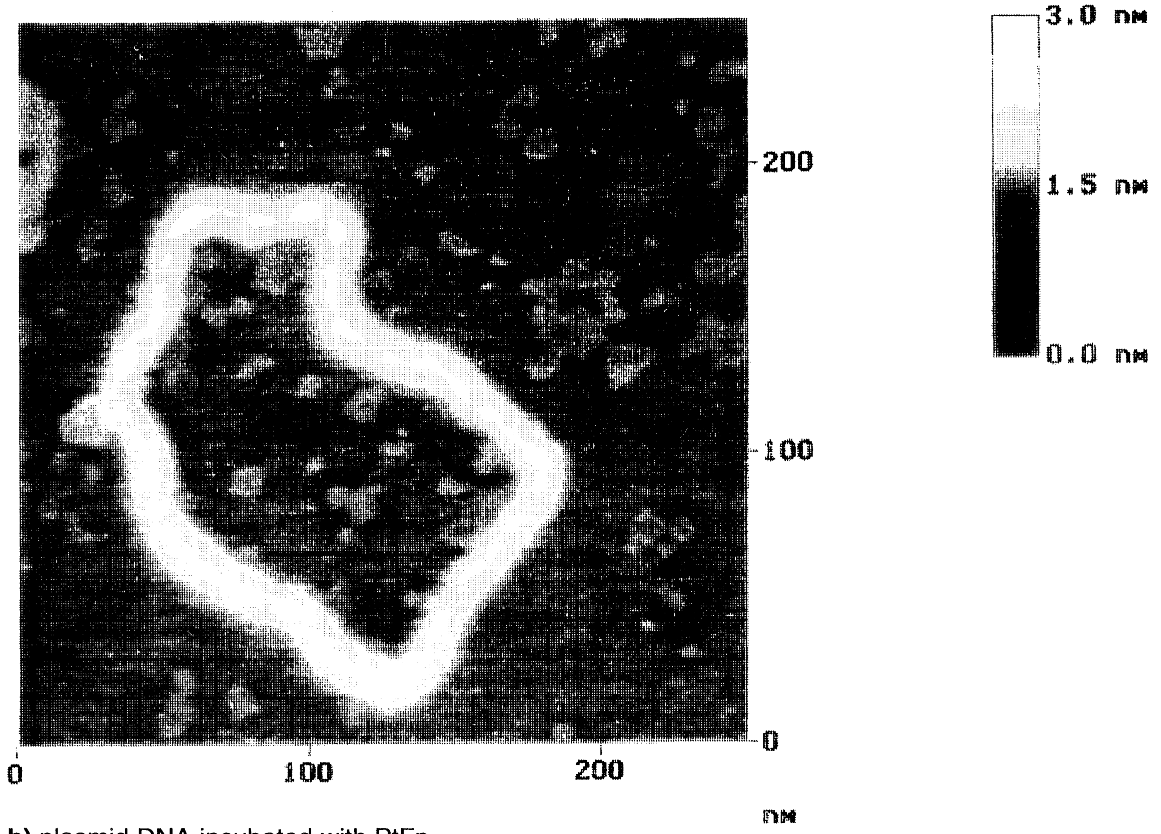

**b)** plasmid DNA incubated with PtFp

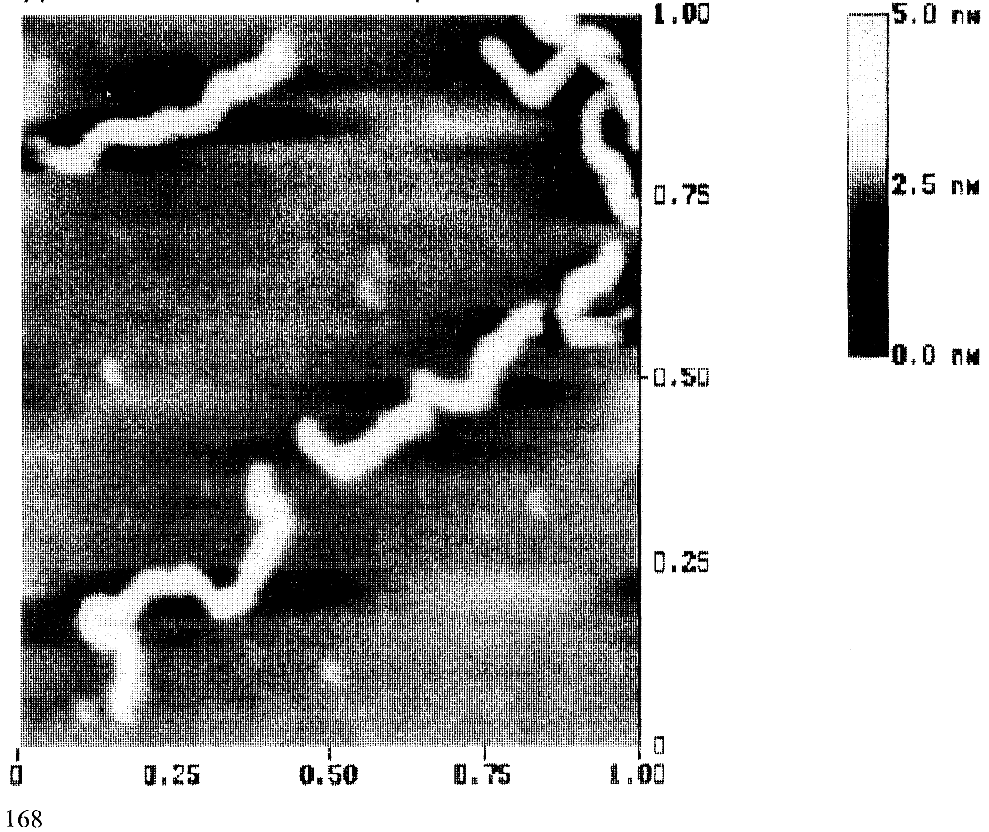

**c)** plasmid DNA incubated with PtBp

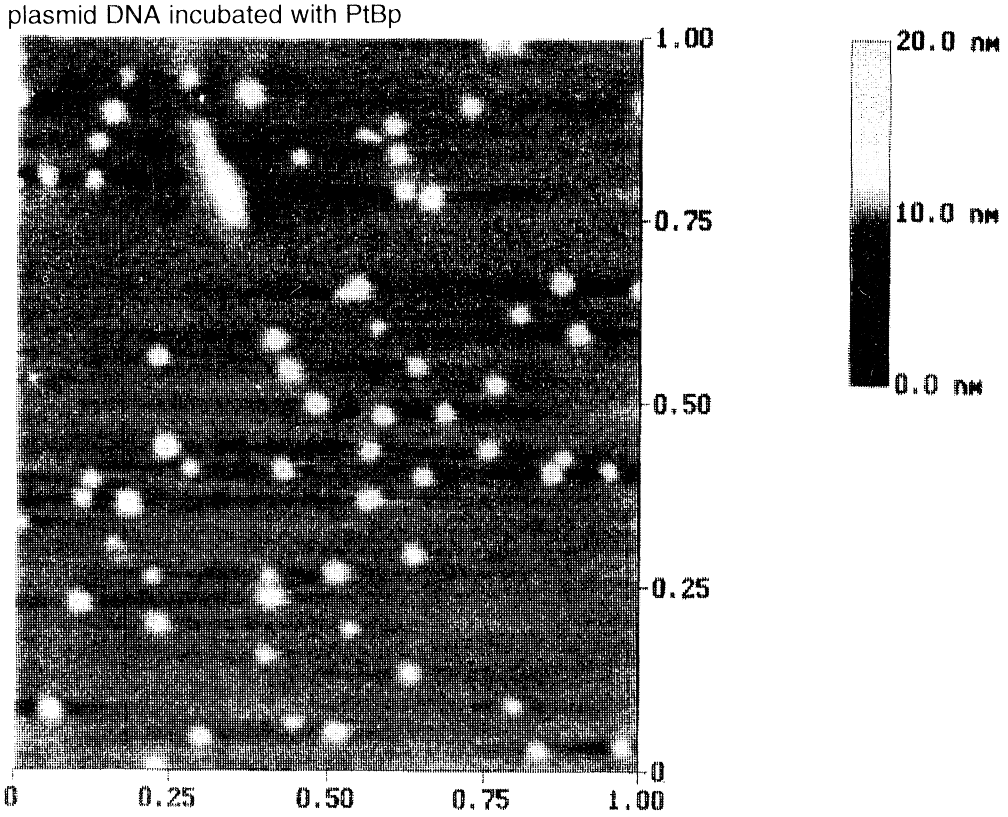

**Table 9:** CD of DNA/HFp compounds at different molar ratio after 24 h incubation.

| Sample | ri | $\Theta_{max}$[a] | $\lambda_{max}$[b] | $\Theta_{min}$[a] | $\lambda_{min}$[b] |
|---|---|---|---|---|---|
| **DNA** | | 8.72 | 274.4 | -10.16 | 245.6 |
| **HFp** | 0.05 | 8.78 | 277.0 | -10.83 | 245.8 |
| | 0.10 | 8.84 | 274.8 | -10.90 | 244.6 |
| | 0.30 | 8.77 | 274.2 | -10.47 | 243.8 |
| | 0.50 | 8.84 | 274.4 | -10.45 | 245.2 |
| **PtFp** | 0.05 | 8.75 | 279.2 | -10.10 | 246.2 |
| | 0.10 | 8.60 | 276.0 | -9.81 | 245.0 |
| | 0.30 | 8.34 | 275.2 | -9.98 | 245.8 |
| | 0.50 | 8.35 | 279.6 | -9.74 | 246.4 |
| **PdFp** | 0.05 | 8.84 | 278.8 | -10.11 | 246.2 |
| | 0.10 | 8.69 | 275.4 | -10.16 | 244.6 |
| | 0.30 | 9.46 | 278.2 | -10.21 | 245.4 |
| | 0.50 | 9.38 | 281.0 | -9.06 | 245.2 |

a: $degree.cm^2.dmol^{-1}.10^3$
b: nm

***Atomic Force Microscopy (TMAFM)***

Figure 3 shows the images corresponding to plasmid pBR322 (a), the same DNA incubated for 24 h with PtFp(b) and incubated for 24 h with PtBp (c). The derivative Pt-Fp modifies the relaxed form of the free plasmid. The closed circular form observed in (a) for the plasmid DNA adopts a supercoiled form, as can be observed in (b). The interaction of the derivative Pt-Bp is more dramatic, the plasmid DNA is converted into oblates. The effects of the free-platinum ligands on the plasmid DNA in the same conditions are not significant.

This is consistent with the results of CD for the same compounds. In both cases the structure of the compounds allows monofunctional covalent interaction between a platinum atom and DNA, but in the case of the Pt-Bp complex the presence of the R group causes additional interaction and the plasmid DNA is strongly modified. Since both complexes have shown their reactivity against DNA, they are excellent candidates to be tested in antitumor and antiviral probes.

***ACKNOWLEDGMENTS:*** *We are grateful to DGICYT Ref. PB94-0922-C02-01 for financial support and to Johnson Matthey for $K_2[PdCl_4]$ and $K_2PtCl_4$ supplied.*

### REFERENCES

1. F.A. French and E.J. Blanz, Jr., *J.Med.Chem.*, **17**, 172 (1974)
2. D.L. Klayman, J.F. Bartosevich, T.S. Griffin, C.J. Mason and J.P. Scovill, *J.Med.Chem.*, **22**, 855 (1979)
3. C. Shipman,Jr., S.H. Smith, J.C. Drach and D.L. Klayman, *Antimicrob. Agents Chemother.*,**19**, 682 (1981)
4. J.P. Scovill, D.L. Klayman and C.F. Franchino, *J.Med.Chem.*, **25**, 1261 (1982)
5. D.L. Klayman, J.P. Scovill, J.F. Bartosevich and J. Bruce, *J.Med.Chem.*, **26**, 35 (1983)
6. J.P. Scovill, D.L. Klayman, C. Lambros, G.E. Childs and J.D. Notsch, *J.Med.Chem.*, **27**, 87 (1984)
7. M. Rahman, M.A. Mridha and M.A. Ali, *Transition Met.Chem.*, **19**, 237 (1994)
8. D.X. West, P.M. Ahrweiler , G. Ertem, J.P. Scovill, D.L. Klayman, J.L. Flippen-Anderson, R. Gilardi, C. George and L.K. Pannell, *Transition Met.Chem.*, **10**, 264 (1985)
9. D.X. West and N.C. Lewis, *Transition Met.Chem.*,**13**, 277 (1988)
10. D.X. West, C.S. Carlson and A.C. Whyte, *Transition Met.Chem.*, **15**, 43 (1990)
11. D.X. West, C.S. Carlson, A.E. Liberta and J.P. Scovill,*Transition Met.Chem.*, **15**, 383 (1990)
12. M.E. Hossain, J. Begum, M.N. Alam, M. Nazimuddin and M.A. Ali, *Transition Met.Chem.*, **18**, 497 (1993)
13. M.A. Ali, K.R. Fernando, D. Palit and M. Nazimuddin, *Transition Met.Chem.*, **20**, 19 (1995)
14. M.E. Hossain, M.N. Alam, M.A. Ali, M. Nazimuddin, F.E. Smith and R.C. Hynes, *Polyhedron*, **15**, 973 (1996)
15. M.A. Ali, K.K. Dey, M. Nazimuddin, F.E. Smith, R.J. Butcher, J. P. Jasinski and J.M. Jasinski, *Polyhedron*, **15**, 3331 (1996)
16. D.X. West, N.M. Kozub and G.A. Bain, *Transition Met. Chem.*, **21**, 52 (1996)
17. D.X. West, J.J. Ingram III, N.M. Kozub, G.A. Bain and A.E. Liberta, *Transition Met. Chem.*, **21**, 213 (1996)
18. M.A. Ali, T.S. Guan, P. Bhattacharjee, R.J. Butcher , J.P. Jasinski and Y. Li, *Transition Met. Chem.*, **21**, 351 (1996)
19. D.X.West, I.S. Billeh, G.A. Bain, J. Valdés-Martínez, K.H. Ebert and S. Hernández-Ortega, *Transition Met. Chem.*, **21**, 573 (1996)
20. M.A. Ali, S.E. Livingstone and D.J.Phillips, *Inorg.Chim.Acta,* **5**, 119 (1970)
21. M.A. Ali, S.E. Livingstone and D.J.Phillips, *Inorg.Chim.Acta,* **5**, 493(1970)
22. M.Das and S.E. Livingstone, *Inorg.Chim.Acta,* **19**, 5 (1976)
23. D.Kovala-Demertzi, A. Domopoulou, M.A. Demertzis, C.P.Raptopoulou and A. Terzis, *Polyhedron*, **13**, 1917 (1994)
24. D.Kovala-Demertzi, A. Domopoulou, M.A. Demertzis, J. Valdés-Martínez, S. Hernández-Ortega, G. Espinosa-Pérez, D.X. West, M.M. Salberg, G.A. Bain and P.D. Bloom, *Polyhedron*, **15**, 2587 (1996)
25. A.C.T. North, D.C. Philips and F.S. Mathews, *Acta Cryst.*, **A24**, 351 (1968)
26. G.M. Sheldrick, *Acta Cryst.*, **A46**, 467 (1990)
27. G.M. Sheldrick, SHELX-93, *A computer program for determination of crystal structure*, University of Göttingen, Germany, 1994
28. *International Tables of X-Ray Crystallography*, Kynoch Press, Vol. 4, (1974)
29. C.N.R. Rao and R. Venkataraghavan, *Spectrochim. Acta,* **18**, 541 (1962)
30. M. Mohan and P. Sharma, *Inorg. Chim. Acta,* **106**, 197 (1985)
31. Z. Gang and L. Feng, *Transition Met. Chem.,* **19**, 567 (1994)
32. M.A. Ali, C.M. Haroon, M. Nazimuddin, S.M. Mahbub-ul-Haque Majumder, M.T.H. Tarafder and M.A. Khair, *Trans. Met. Chem.,* **17**, 133 (1992)
33. M. Nazimuddin, M.A. Ali and F.E. Smith, *Trans. Met. Chem.,* **16**, 528 (1991)
34. K.L. Busch, G.L. Glish, K.L. Busch, G.L. Glish and S.A. Mcluckey, *Techniques and applications of tandem Mass Spectrometry,* VCH Publishers, New York (1988)
35. J.P. Macquet and J.L. Butour, *Eur. J. Biochem.,* **83**, 375 (1978)
36. J.P. Macquet and J.L. Butour, *Biochimie,* **60**, 901 (1978)

**Received: March 26, 1998 - Accepted: April 20, 1998 - Received in revised camera-ready format: May 7, 1998**

